# Satisfaction of clients with the services of an outpatient pharmacy at a university hospital in northwestern Ethiopia: a cross-sectional study

**DOI:** 10.1186/s12913-015-0900-6

**Published:** 2015-06-11

**Authors:** Abdrrahman Shemsu Surur, Fitsum Sebsibe Teni, Genet Girmay, Elsabet Moges, Meseret Tesfa, Messele Abraha

**Affiliations:** Department of Pharmaceutical Chemistry, College of Medicine and Health Sciences, University of Gondar, Gondar, Ethiopia; Department of Pharmaceutics and Social Pharmacy, College of Medicine and Health Sciences, University of Gondar, Gondar, Ethiopia

**Keywords:** Satisfaction, Pharmacy, Clients, Services, Ethiopia, Gondar University Referral Hospital

## Abstract

**Background:**

Evaluation of patient/client satisfaction with pharmacy services as a crucial part of the health services through appropriate studies is important. This will help identify specific areas of the service which need improvement in realizing high quality pharmacy services in general and enhance the positive changes in the current pharmaceutical services provision in Ethiopia. The current study aimed at assessing the level of client satisfaction with the services of the outpatient pharmacy of Gondar University Referral Hospital (GURH) in northwestern Ethiopia.

**Methods:**

An institution-based cross-sectional study was conducted involving 400 clients who had prescriptions/orders filled at the outpatient pharmacy of the hospital during the period of 5th to 25th of November 2013. The data on the level of satisfaction of clients with the services of the outpatient pharmacy in the hospital was collected using a structured interview guide adopted from an instrument translated into Amharic and validated. The data collected was entered into and analyzed using Statistical Packages for Social Sciences (SPSS) version 16.

**Results:**

The overall mean score the respondents gave to satisfaction with the pharmaceutical services was 2.48 out of a maximum of 5.00 score. The mean scores for all the individual parameters rated were less than 3.00. Maximum mean scores were given for parameters asking about the promptness of prescription medication service (2.99), and professionalism of the pharmacy staff (2.96) with the lowest being scored for information given to clients about the storage of medication (1.25), and explanations of possible side effects (1.27). Clients who were served free of fee recorded significantly higher level of satisfaction than those who paid. Higher levels of satisfaction were also reported among illiterates, older adults and those with no job compared to those with higher education, merchants and government employees.

**Conclusions:**

This study showed that the overall mean satisfaction level of clients of the outpatient pharmacy was low and it differed among different socio-demographic characteristics. Further research in to the reasons behind the low satisfaction should be done to provide appropriate solutions to improve the service.

## Background

Good pharmacy practice (GPP) was defined by joint International Pharmaceutical Federation (FIP)/World Health Organization (WHO) guidelines on GPP as the practice of pharmacy that responds to the needs of the people who use the pharmacists’ services by providing optimal, evidence-based care. According to the guidelines it required that a pharmacist’s priority be the welfare of patients; helping patients make the best use of medicines as a main activity; contributing to rational and economic prescribing and dispensing; and that pharmacy services objectives should be relevant and properly communicated to patients [1].

American society of hospital pharmacists defined the mission of the pharmacist, with some modification on the definition by Hepler and Strand, 1990 as the provision of pharmaceutical care which in turn was defined as the direct, responsible provision of medication-related care for the purpose of achieving definite outcomes that improve a patient’s quality of life [2, 3].

In order to ensure the implementation of GPP with the current philosophy of pharmaceutical care, its quality should be assessed. In this process quality could be seen from three aspects including structure, process and outcome of the services provided [4]. Structure includes resources, care providers, settings in which the services are provided and their organization. The process component describes activities performed in the delivery of care and its quality is assessed from the perspective of scientific and societal value. On the other hand outcome refers to the results of care or their effects on the individual’s health condition and it is considered by many that its measurement serves as valid indicators of quality. Satisfaction is defined as and assumed to entail cognitive evaluation and an emotional reaction to the structure, process, and outcome of services [5].

Patient satisfaction has been described to improve other treatment outcomes in addition to being a goal of treatment by itself [6]. So, evaluation of patient/client satisfaction with pharmacy services as a crucial part of the health services through appropriate studies is important. This will be helpful in identifying specific areas of the service which need improvement in realizing high quality pharmacy services in general and also for enhancing the positive changes in the current pharmaceutical services provision in Ethiopia. This in turn will provide information to advance pharmacy services in a way that it can optimize services to ensure clients’ health outcomes by addressing their concerns and their needs from the services.

In the measurement of the level of satisfaction of patients/clients with the services of pharmacies different instruments have been used [7–10]. Different studies have been conducted globally on the satisfaction of patients with the pharmacy services rendered to them [11]. From the various studies which applied a variety of approaches, different findings have been reported. Among these, a study done in Brazil reported satisfaction of clients of private community pharmacy services which provided pharmaceutical care in comparison with those not providing it [12]. Other studies in Qatar and Saudi Arabia reported on the levels of satisfaction of clients with the services of community and hospital pharmacies respectively [13, 14].

Another study done in Botswana reported satisfaction of clients with quality of a primary health care facility which contained pharmacy services as one part [15]. Studies in Spain which focused on outpatient and community pharmacies also reported findings on the levels of satisfaction of patients with the services in each setting [16, 17]. Another study in Pakistan reported satisfaction levels of clients of outpatient departments of a health facility in Islamabad, which assessed pharmacy services as a component [18].

Pharmacy practice in Ethiopia has traditionally involved product oriented approach majorly. The different practice areas in the country include health institution pharmacy, community medicine retail outlets, the pharmaceuticals supply system, manufacturing establishments, medicines regulatory body and academic areas among others. In recent times there have been moves toward a major shift in the direction of patient oriented pharmacy services. In line with this a change in the curriculum, which was previously product focused, to a five years clinically oriented pharmacy education curriculum has been instituted. In the year 2013, through this curriculum 426 pharmacists graduated and in relation to this there are efforts in different hospitals of the nation especially in university hospitals toward provision of clinical pharmacy services through involvement in patient care in wards, other hospital pharmacy sections [19–22].

In Ethiopia, few studies on the satisfaction of clients/patients with the general health care services in different health institutions have been conducted which did not emphasize pharmacy services [23–26]. One study done in Addis Ababa assessed the quality of pharmaceutical services in governmental hospitals, assessing satisfaction of clients with the services as one component [27]. As evident from the scarce documentation of the opinion of patients towards pharmacy services in different parts of Ethiopia, conducting studies answering this question is important. The current study aimed at assessing the level of client satisfaction with the services of the outpatient pharmacy and its variation with socio-demographic characteristics in Gondar University Referral Hospital (GURH), northwestern Ethiopia.

## Methods

### Study setting and design

An institution-based cross-sectional study was conducted at GURH, located in the northwestern Ethiopian town of Gondar. The institution is a referral and teaching hospital with a catchment population of 5 million. GURH has more than 400 beds capacity and provides its services in various departments including internal medicine, surgery, pediatrics, gynecology and obstetrics, dermatology, dentistry, ophthalmology, pharmacy (outpatient, inpatient, antiretroviral, and emergency), medical laboratory and others (GURH Statistics and Information Office: Annual Report on Health Services and Employees, Gondar, Ethiopia 2013, unpublished).

### Sampling

The sample size of participants to be included in this study was determined using single mean formula: [N = (Z_1 ‐ α_)^2^ × *σ*^2^/SE^2^] considering the standard deviation (σ) of mean satisfaction level to be 0.5 based on the finding from the pretest done on 41 interview encounters. The margin of error (standard error (SE)) was set to be 0.05 with 95 % confidence interval (CI) [28]. With an added contingency of 5 % for non response and inappropriate responses, the final sample was calculated to be 405 clients.

In selecting participants of the study, adult clients (18 years or older) who were willing and had their prescript/orders filled at the outpatient pharmacy of the hospital were included in the study. The study was conducted during the period of 5th to 25th of November 2013. The average daily client flow to the pharmacy was estimated to be about 100 and the number of clients to be interviewed during the 21 days of data collection was 21. By dividing the daily client flow to the pharmacy with the number of clients to be surveyed per day, every fifth client was approached the interview. The first client was selected daily through drawing a number from 1 up to 5 and continuing with every fifth number until the daily sample limit was reached.

### Data collection instrument and process

The data on the level of satisfaction of clients with the services of the outpatient pharmacy in university hospital was collected using a structured interview guide. The guide was adopted from an instrument employed by Eshetu and Gedif, 2011 in a study done in governmental hospitals of Addis Ababa to assess the level of satisfaction of clients with the services of the pharmacies in the hospitals. The instrument was assessed for its reliability in the cited study and had Cronbach’s alpha of 0.9 [27].

The instrument contained two sections, one section which focused on the socio-demographic profile of respondents and another on the level of satisfaction of the clients with the services provided in the pharmacy. The section focused on the level of satisfaction of clients contained twenty one five-point Likert scale items. On the scale “1” stood for a rating of the item as “very low” while “2”, “3”, “4” and “5” stood for “low”, “moderate”, “High” and “Very high” in that order. Based on this, the mean level of satisfaction of clients was calculated by averaging their ratings for the 21 parameters of measuring satisfaction. The resulting mean was interpreted by considering the closest Lickert scale to it.

The data collection instrument was pretested on 41 clients, which were excluded from the final analysis, before the commencement of actual data collection process.

The data was collected by four of the principal investigators through interviewing clients after they had their prescriptions/orders filled in the pharmacy. The investigators who collected the data were properly trained on the instrument and ways of approaching clients and securing their permission for interview prior the data collection process.

### Data entry and analysis

The data collected was entered into and analyzed using Statistical Packages for Social Sciences (SPSS) version 16. In the study socio-demographic and satisfaction level of the clients were described using frequencies, percentage, mean and standard deviation. Independent *t* test was employed to assess the difference in satisfaction of clients between sex, payment and patronage. On the other hand one way ANOVA was used to check the difference among age groups and educational status of the clients with regard to level of satisfaction with the services provided in the pharmacy. In doing the different analyses 95 % CI and p value of 0.05 were used for deciding statistical significance of differences observed [29].

### Ethics

This study was approved by the ethical review committee of the school of pharmacy, University of Gondar. In addition permission was secured from the hospital and the pharmacy administrators to proceed with the study. Each of the clients were provided with explanations on the purpose of the study and asked for their consent to participate in the study. Only when they were willing to proceed with the interview were they involved in the study. In addition, patient identifiers were not used in the study and the data collected was used by the investigators only for the purpose of the study.

## Results

### Socio-demographic characteristics of respondents

In this study out of the total questionnaires/interview guides of sample of 405 clients who were interviewed 400 were included in the analysis, and five encounters were excluded due to incompleteness making the response rate 98.8 %. Most of the respondents were male (60.2 %), married (68.2 %) and Orthodox Christians (79.2 %). Among the respondents, those in the age group of 30 to 39 years old constituted the highest proportion (35.5 %) followed by those in the age group of 18 to 29 years of age (31.5 %) (Table 1).

**Table 1:** Socio-demographic characteristics of respondents, GURH, 2013

Variable		Frequency (%)
Sex	Male	241 (60.2)
	Female	159 (39.8)
Age (years)	18−29	126 (31.5)
	30−39	142 (35.5)
	40−49	73 (18.2)
	50−59	41 (10.2)
	60+	18 (4.5)
Marital status	Single	102 (25.5)
	Married	273 (68.2)
	Divorced	9 (2.2)
	Widowed	16 (4.0)
Educational status	Not able to read and write	56 (14.0)
	Primary school	33 (8.2)
	Junior secondary school	31 (7.8)
	Secondary school	103 (25.8)
	Higher education	177 (44.2)
Occupation	Employee of government institution	108 (27.0)
	Employee of private institution	60 (15.0)
	Farmer	61 (15.2)
	Merchant	62 (15.5)
	No job	105 (26.2)
	Others	4 (1.0)
Religion	Orthodox Christianity	317 (79.2)
	Islam	79 (19.8)
	Protestantism	4 (1.0)
Locality	Gondar Town	292 (73)
	Out of Gondar Town	108 (27)
Patronage	First time visit	94 (23.5)
	Repeat visit	306 (76.5)
Service sought for	Self	264 (66)
	Others (family/relatives)	136 (34)
Payment status	Free	83 (20.8)
	Paying	317 (79.2)

Respondents who were from Gondar town (73.0 %), those taking medications for themselves (66.0 %) and those who paid for the medications (79.2 %) constituted the highest proportion in their respective categories (Table 1).

As regards to the educational status of respondents the most frequent had a status of higher education (44.2 %) followed by those completing secondary education (25.8 %). Among the respondents employees of government institutions (27.0 %) and those with no job (26.2 %) constituted majority of the respondents (Table 1).

### Satisfaction level of clients with the services of the pharmacy

In the present study of the satisfaction level of pharmacy clients towards the pharmaceutical services, all the 400 respondents gave their responses to all the twenty-one parameters they were asked to rate. The overall mean score the respondents gave to satisfaction with the pharmaceutical services was 2.48 out of a maximum of 5.00 score (Table 2).

**Table 2:** The proportions and mean of satisfaction scores of respondents with the pharmaceutical services, GURH, 2013

Variable	Very low (%)	Low (%)	Moderate (%)	High (%)	Very high (%)	Mean (SD)
The pharmacy professional’s interest in your health	24 (6.0)	66 (16.5)	224 (56.0)	83 (20.8)	3 (0.8)	2.94 (0.80)
The professionalism of all the pharmacy staff	14 (3.5)	64 (16.0)	244 (61.0)	78 (19.5)	0 (0.0)	2.96 (0.70)
The courtesy and respect shown to you by the pharmacy staff	12 (3.0)	86 (21.5)	218 (54.5)	84 (21.0)	0 (0.0)	2.94 (0.74)
The privacy of your conversations with the pharmacist	12 (3.0)	104 (26.0)	234 (58.5)	50 (12.5)	0 (0.0)	2.80 (0.68)
How well the pharmacist explains possible side effects	326 (81.5)	48 (12.0)	19 (4.8)	7 (1.8)	0 (0.0)	1.27 (0.63)
The promptness of prescription medication service	22 (5.5)	79 (19.8)	181 (45.2)	118 (29.5)	0 (0.0)	2.99 (0.84)
The care the pharmacy professional takes while supplying your medications	12 (3.0)	86 (21.5)	230 (57.5)	70 (17.5)	2 (0.5)	2.91 (0.72)
The fairness of cost of medications in the pharmacy	24 (6.0)	128 (32.0)	174 (43.5)	72 (18)	2 (0.5)	2.75 (0.84)
The amount of time the pharmacy professional spends with you	45 (11.2)	134 (33.5)	183 (45.8)	37 (9.2)	1 (0.2)	2.54 (0.82)
The clarity of the pharmacy professional’s instructions about how to take your medication	27 (6.8)	96 (24.0)	228 (57.0)	48 (12.0)	1 (0.2)	2.75 (0.76)
The information the pharmacist gives you about the proper storage of your medication	332 (83.0)	43 (10.8)	19 (4.8)	6 (1.5)	0 (0.0)	1.25 (0.61)
How well the pharmacy professional answers your questions	20 (5.0)	88 (22.0)	232 (58.0)	59 (14.8)	1 (0.2)	2.83 (0.74)
The information the pharmacy professional gives you about the results you can expect from your medication therapy	238 (59.5)	90 (22.5)	60 (15.0)	12 (3.0)	0 (0.0)	1.62 (0.85)
The way your pharmacist works together with your doctor to make sure your medications are the best for you	241 (60.2)	96 (24.0)	59 (14.8)	4 (1.0)	0 (0.0)	1.56 (0.78)
The amount of time you spend waiting for your prescription to be filled	27 (6.8)	119 (29.8)	206 (51.5)	47 (11.8)	1 (0.2)	2.69 (0.78)
The availability of medications that are prescribed to you in the pharmacy	33 (8.2)	120 (30.0)	166 (41.5)	80 (20.0)	1 (0.2)	2.74 (0.88)
The clarity of the label on the medication supplied to you	29 (7.2)	102 (25.5)	219 (54.8)	49 (12.2)	1 (0.2)	2.73 (0.78)
Your feelings of the quality of medication dispensed to you	26 (6.5)	102 (25.5)	184 (46.0)	87 (21.8)	1 (0.2)	2.84 (0.85)
The overall cleanliness and comfort of the waiting area	252 (63.0)	95 (23.8)	42 (10.5)	11 (2.8)	0 (0.0)	1.53 (0.79)
The location of the pharmacy relative to other service areas	42 (10.5)	82 (20.5)	161 (40.2)	113 (28.2)	2 (0.5)	2.88 (0.96)
Your pharmacy services overall	32 (8.0)	118 (29.5)	190 (47.5)	59 (14.8)	1 (0.2)	2.70 (0.83)

Looking at the mean scores for the individual parameter rated, all of them were less than 3.00. Among the mean scores the maximum scores were given for parameters including “the promptness of prescription medication service” (2.99), and “the professionalism of all the pharmacy staff” (2.96). On the other hand, the parameters rated lowest included “the information the pharmacist gives you about the proper storage of your medication” (1.25), and “how well the pharmacist explains possible side effects” (1.27) (Table 2).

Among the parameters rated by the respondents each were rated to be of ‘very high (5.00)’ level of satisfaction only by less than 1 % of the total respondents. In addition, in nearly half (42.9 %) the parameters none of the respondents rated the parameters as ‘very high (5.00)’ (Table 2).

### Difference in satisfaction level among respondents

The difference in the mean satisfaction levels of the clients of the pharmacy involved in the study was checked with respect to socio-demographic characteristics. Based on the independent samples *t* test performed on sections of the socio-demographic variables, payment status showed statistically significant difference. Clients served in the pharmacy with no payment required of them were showed to have had higher level of mean satisfaction compared to clients who paid for the services received (Table 3).

**Table 3:** Test of statistical significance (independent samples *t*-test) of variation in the mean satisfaction level of clients by socio-demographic characteristics, GURH, 2013

Variable	Mean (SD)	*p*-value
Sex		
Male	2.49 (.304)	.848
Female	2.48 (.363)	
Locality		
Gondar Town	2.47 (.326)	.129
Out of Gondar Town	2.53 (.330)	
Patronage		
First time visit	2.47 (.301)	.617
Repeat visit	2.49 (.336)	
Payment Status		
Free/do not pay	2.65 (.268)	<.001*
Paying	2.44 (.329)	
Service sought for		
Self	2.49 (.323)	.572
Others (family/relatives)	2.47 (.338)	

* p-value <0.05

In the one way ANOVA test performed on different socio-demographic characteristics of clients in the study; age, educational status and occupational status were found to have statistically significant differences. The Post Hoc test (Tukey HSD) revealed that, the age groups 50 to 59 years of age (*p*-value = .016) and those 60 years or older (*p*-value = 0.030) had higher mean satisfaction levels compared to those in the age group of 18 to 29 years of age.

On the other hand a similar Post Hoc test on educational status uncovered that clients in the group with higher education had a statistically significant lower mean satisfaction level compared to illiterate clients (*p*-value = .001). With regard to occupational status, farmers showed higher level of mean satisfaction compared to government employees (*p*-value = 0.004) and merchants (*p*-value = 0.014) based on the Post Hoc test. In the same group clients with no job were found to be more satisfied with the services they received compared to government employees (*p*-value = 0.034) (Table 4).

**Table 4:** Test of statistical significance (One way ANOVA test) of the variation in the mean satisfaction level of clients by socio-demographic characteristics, GURH, 2013

Variable	Mean (SD)	*p*-value
Age (in years)		
18−29	2.42 (.351)	.001^*^
30−39	2.46 (.330)	
40−49	2.54 (.284)	
50−59	2.60 (.286)	
60+	2.66 (.328)	
Marital status		
Single	2.43 (.379)	.062
Married	2.50 (.308)	
Divorced	2.41 (.228)	
Widowed	2.63 (.282)	
Educational status		
Illiterate	2.62 (.310)	.003^*^
Primary school	2.55 (.400)	
Junior secondary school	2.51 (.414)	
High school	2.48 (.316)	
Higher education	2.43 (.296)	
Occupational status		
Government employee	2.41 (.334)	<.001^*^
Private company employee	2.44 (.324)	
Farmer	2.60 (.315)	
Merchant	2.43 (.307)	
No job	2.56 (.318)	
Other	2.27 (.137)	
Religion		
Orthodox Christianity	2.49 (.326)	.079
Islam	2.49 (.331)	
Protestantism	2.12 (.208)	

* p-value <0.05

## Discussion

In the present study the mean level of satisfaction was found to be low as it fell below the “moderate” level in the five point Likert scale. The reported mean level of satisfaction was lower compared to findings in Spain and in Portugal on community pharmacies which reported high level of satisfaction among clients [16, 30]. This could be attributed to the difference in the development levels of the countries generally and their pharmacy services particularly. In addition the finding on the present study was found to be lower than that of a study in Botswana which assessed clients’ satisfaction with pharmacy services as part of overall health services and reported a high level of satisfaction [15]. The difference in the level of satisfaction reported by clients compared to the situations in the studies cited above indicates that there is a gap in system and service related aspects of pharmacy services in the institution.

The low level of satisfaction on the side of clients in this study was comparable to the finding from a study done in Addis Ababa, on pharmacies of governmental hospitals which reported a mean level of overall satisfaction of 2.7 [27]. Similarly lower than average level of satisfaction was reported for medication counseling by pharmacists before intervention at a psychiatric hospital in Nigeria [31].

The parameters “the promptness of prescription medication service”, and “the professionalism of all the pharmacy staff” were among those rated highest in the current study. Different to this pharmacist’s skills and confidentiality, and assistance to patients were rated highest as reported by a study at an outpatient pharmacy in Spain [16].

On the other hand the lowest rated parameters in the present study were “the information the pharmacist gives you about the proper storage of your medication”, and “how well the pharmacist explains possible side effects” which showed similar finding to the study in Addis Ababa [27]. This indicates that services related to specific medications, especially their availability, information on side effects, storage, expected results from the medications and other were responsible for the lower level of satisfaction. As was similar in the study done in Addis Ababa, these areas of pharmacy service are with problems [27]. The medication related services which were sources of low satisfaction among clients is supported by a finding from a study in the northwestern Ethiopian town of Bahir Dar. In this cited study, done through observational method, it was reported that only 32.8 % of the counseling in the outpatient pharmacies of the participating health institutions was considered satisfactory [32].

In this study the results of independent samples *t* test showed that clients who paid for the services in the pharmacy had a statistically significant lower level of satisfaction compared to those served with no payment. A similar finding was reported in the study done in Addis Ababa [27]. In Ethiopia there is a system of waiving people with very little income from fees required to get basic health services, provision of medicines being one of them. So, the higher rating of the services in the pharmacy by those who do not pay could be associated to their tolerance to other factors which could have affected their rating because of the help they got through waiver of payment.

One way ANOVA performed also on socio-demographic characteristics showed statistically significant differences among different age groups, educational levels and occupational status of clients in the study. The higher level of satisfaction reported among elderly clients compared to the young ones and the higher level of satisfaction among illiterate clients than in those having higher education were interrelated. This was evident in the chi square test performed which revealed that level of education was different in a statistically significant manner among different age groups, with younger clients being more educated. The higher level of satisfaction among the illiterate and elderly clients could be associated to lesser awareness on the importance of details of the functions in the pharmacy and the services they deserved to get, so overrating it.

Differences in satisfaction among different age groups was also reported by the study done in Portugal [30]. In contrast to the present study higher mean satisfaction levels among clients of university level education compared to those of lower educational levels, in different aspects of services, were reported by a study done in Riyadh, Saudi Arabia [14].

Satisfaction levels were also found to be statistically different among different occupational groups. Among these, farmers and those without job tended to be more satisfied compared to merchants and government employees. The occupational groups were also different depending on educational status in a statistically significant manner based on the chi square tests done. This showed, similarly to the case of satisfaction level with age groups above, those with no job and farmers were of lower educational status. The higher level of satisfaction could be similar to the above mentioned ones. Unlike this, government employees and merchants who were relatively more educated and with better awareness of what to expect from the services rated their satisfaction lower.

### Limitation

In this study an important limitation is the fact that the specific services clients got in the pharmacy were not assessed. This hindered the study’s ability to assess the level of satisfaction of clients in relation to the kind of services received. This would have helped suggest specific services to focus on for future studies.

## Conclusions

This study showed that the overall mean satisfaction level of clients of the outpatient pharmacy was low. Mean satisfaction level was found to be different in a statistically significant manner between/among different groups including age, educational status, occupational status and payment status. The low satisfaction levels reported should be further studied through qualitative studies to find appropriate solutions in solving the problems. The hospital should institute better service provision system in relation to availing medicines, continuing professional development to professionals to improve the satisfaction of clients with the services provided in the pharmacy.

## Abbreviations

ANOVA: Analysis of variance
CI: Confidence interval
FIP: International Pharmaceutical Federation
GURH: Gondar University Referral Hospital
GPP: Good pharmacy practice
SPSS: Statistical packages for social sciences
WHO: World Health Organization
